# Recurrence in Oral Leukoplakia: A Systematic Review and Meta-analysis

**DOI:** 10.1177/00220345241266519

**Published:** 2024-09-18

**Authors:** B.P. Bhattarai, A.K. Singh, R.P. Singh, R. Chaulagain, T.M. Søland, B. Hasséus, D. Sapkota

**Affiliations:** 1 Department of Oral Biology, Faculty of Dentistry, University of Oslo, Oslo, Norway; 2 Department of Oral and Maxillofacial Surgery, Maharajgunj Medical Campus, Institute of Medicine, Tribhuvan University Teaching Hospital, Kathmandu, Nepal; 3 Department of Oral and Maxillofacial Surgery, University Hospital Southampton, Southampton, UK; 4 Department of Oral Biology, Chitwan Medical College, Bharatpur, Nepal; 5 Department of Pathology, Oslo University Hospital, Oslo, Norway; 6 Department of Oral Medicine and Pathology, Institute of Odontology, The Sahlgrenska Academy, University of Gothenburg, Gothenburg, Sweden

**Keywords:** oral potentially malignant disorders, risk factors, malignant transformation, surgical excision, laser therapy, evidence-based practice

## Abstract

The management of oral leukoplakia (OL) is challenging because of a high risk for recurrence and malignant transformation (MT), and recurrent OL is associated with a higher risk of MT than nonrecurrent OL. The present meta-analysis aimed to examine the association between OL recurrence and surgical techniques used for their management as well as their clinicopathological factors. Electronic searches were conducted in EMBASE, PubMed, Scopus, and Web of Science to retrieve studies reporting OL recurrence after surgery. The pooled proportion of OL recurrence after surgical excision was estimated. Subgroup analyses were conducted based on the surgical technique, data type, grades of epithelial dysplasia, anatomical subsites, clinical type and size of the lesion, surgical margin, and risk habits. Meta-regression analyses were conducted to identify the association between age, sex, and follow-up duration and OL recurrence. The risk of MT based on the recurrence status was also estimated. A network meta-analysis was performed to determine the surgical modality associated with the least OL recurrence. Eighty studies with a total of 7,614 samples and various surgical modalities (laser-based techniques, conventional scalpel surgery, cryosurgery, and photodynamic therapy) were included in the meta-analysis. A pooled proportion of recurrence of 22% was observed. Laser-based surgeries resulted in fewer OL recurrences than other surgical modalities, and the combination of laser excision and vaporization was identified to be the best treatment approach. OL in the retromolar area and multiple sites, nonhomogeneous OL, advanced age, female sex, inadequate surgical margin, retrospective data, and betel quid chewing habit were significantly associated with higher OL recurrence. Recurrent OL showed a 7.39 times higher risk of MT than nonrecurrent OL. These results suggest that the combination of laser excision and vaporization might reduce OL recurrence. Furthermore, OL in older patients, females, and nonhomogeneous OL need close monitoring after any surgical therapy.

## Introduction

Oral leukoplakia (OL) is an oral potentially malignant disorder defined as “a predominantly white plaque of questionable risk having excluded (other) known diseases or disorders that carry no increased risk for cancer” (Warnakulasuriya et al. 2021). It affects an estimated 4.1% of the world’s population, with a huge global variation in prevalence (Mello et al. 2018). Buccal mucosa, tongue, and floor of the mouth are the most frequent intraoral sites for OL. Clinically, OL can be classified as homogeneous (HOL) or nonhomogeneous (NHOL) subtypes. The most feared consequence of OL is malignant transformation (MT) to oral squamous cell carcinoma (OSCC). The MT rate differs with respect to studies, and pooled MT rates of 3.5% (Warnakulasuriya and Ariyawardana 2016) and 9.7% (Pinto et al. 2020) to 9.8% (Aguirre-Urizar et al. 2021) have been reported previously. Several histopathological features, such as epithelial dysplasia, and clinical factors, such as subtype and size of OL and patient age, may influence the MT of OL (van der Waal 2009). Interestingly, a recurrent OL (lesion appearing at the same site after complete excision) has been suggested to have a high risk of MT independent of other clinicopathological factors. In the study by Yang et al. (2010), recurrent OL lesions were reported to have a relative risk of 9.40 for MT compared to the nonrecurrent lesions (Yang et al. 2010). Likewise, Yao et al. (2022) reported 3.14 times higher odds of MT for recurrent OL than nonrecurrent OL. Therefore, early detection and appropriate management of OL are warranted to prevent the recurrence and potential progression to OSCC.

Several chemopreventive and surgical strategies have been employed to treat OL, with surgical interventions being the mainstay of treatment. The most common surgical modalities include conventional scalpel surgery, laser excision, laser vaporization/ablation, cryosurgery, and photodynamic therapy (PDT) (Nadeau and Kerr 2018). However, the effectiveness of various surgical approaches in preventing recurrences remains a matter of both scientific interest and clinical significance. Up to 49% of OL lesions have been reported to recur within 5 y after conventional scalpel surgery (Sundberg et al. 2019). A pooled recurrence rate of 16.5% has been reported for OL after treatment with laser-based surgeries (de Pauli Paglioni et al. 2020). The recurrence rate of OL after cryosurgery has been reported from 25% (Kawczyk-Krupka et al. 2012) to 70% (Ishii et al. 2004). Furthermore, PDT has been found to be associated with up to a 38% recurrence rate (Yao et al. 2022). However, variables like the site and size of the lesion, clinical subtypes, epithelial dysplasia, and risk habits (smoking, alcoholism, betel quid chewing) might affect the recurrence rate among patients treated with similar surgical therapy.

Previous studies, including meta-analyses, have shown that the choice of surgical excision method and clinical/histological features of OL could influence the risk of OL recurrence. However, such studies have mainly focused on a single or few surgical modalities. For instance, de Pauli Paglioni et al. (2020) analyzed the effect of laser-based techniques on the recurrence of OL, and Zhang et al. (2023) investigated the influence of PDT on OL recurrence. Furthermore, the influence of clinicopathological factors on the recurrence of OL needs a comprehensive investigation. This underscores the need for a comprehensive and updated systematic review and meta-analysis. This study aims to provide an evidence-based, comprehensive analysis of the recurrence of OL after various surgical excision approaches. By analyzing the available literature, we seek to present data on the relative effectiveness of diverse surgical interventions and identify clinicopathological factors that may influence the risk of recurrence. We specifically aim to answer the following questions: how does OL recurrence differ among various surgical treatments, such as laser excision and/or ablation, scalpel excision, cryotherapy, and PDT? Does recurrence vary based on the anatomical subsite, size, clinical type, surgical margin of OL, and the degree of epithelial dysplasia? Are patient-related factors, such as age, sex, duration of follow-up, and risk habits associated with OL recurrence? Is recurrence a risk factor for MT of OL?

## Methods

### Literature Search

A protocol for the study was registered in the International Prospective Register of Systematic Reviews, PROSPERO (CRD42023445985). A systematic review was performed on all publications concerning the recurrence of OL after various surgical treatments following the Preferred Reported Items for Systematic Reviews and Meta-Analyses guidelines. The PICO criteria for study selection were as follows: P, patients with oral leukoplakia; I, surgical treatment; C, different surgical treatments; and O, recurrence. A literature search was conducted using EMBASE, PubMed, Scopus, and Web of Science. All studies published in English from database inception to April 2024 were considered. In addition, the reference lists of all eligible articles were searched for additional studies not initially identified through the database search. Details of the search strategies are presented in Appendix Methods.

### Study Selection

Two independent reviewers (A.K.S. and B.P.B.) conducted the study selection. All studies reporting the recurrence of OL after surgical treatment involving patients in real clinical settings (not in vitro studies) were selected. The exclusion criteria were studies that included data only on MT or those using therapies other than surgical excision (e.g., vitamin A), studies that involved only proliferative verrucous leukoplakia, case reports, journal or conference abstracts (structured or unstructured), reviews, letters to the editors, and book chapters. Furthermore, when studies reported treatment outcomes for oral potentially malignant disorders (including proliferative verrucous leukoplakia), we extracted the data specific only to oral leukoplakia. Any discrepancies on the inclusion/exclusion of certain studies were resolved by discussion or having a third opinion (D.S.).

### Data Extraction and Analysis

Data were systematically extracted from the included studies after a qualitative assessment based on the above PICO criteria. Data were recorded by 1 reviewer (B.P.B.) and confirmed by another reviewer (A.K.S.). Data on the following items/variables were obtained: author, country, study design, objectives of the study, data sources, main findings, type of surgical treatment, population characteristics, site of the lesion and recurrence, recurrence rates, clinical type of lesion, follow-up period, and other reported outcomes.

### Risk of Bias Assessment

Quality Assessment Tool for Observational Cohort and Cross-Sectional Studies was used to assess the risk of bias (RoB) for observational and cross-sectional studies (Study Quality Assessment Tools 2021). For randomized controlled clinical studies, the Cochrane Handbook for Systematic Reviews of Interventions version 5.2.0 was used (Higgins et al. 2017). Two reviewers (A.K.S. and R.C.) independently assessed the risk of bias, and any disagreement concerning any entry was resolved by discussion. ROBVIS, a web-based service based on RStudio, was used to create the graphical illustrations of the RoB (McGuinness and Higgins 2021).

### Statistical Analysis

RStudio and OpenMeta [Analyst] software were used for statistical analysis. Stata version 18.0 (StataCorp) was used to acquire graphical results. A narrative synthesis was performed to summarize the characteristics of the included studies. A random-effects meta-analysis of proportion was performed to report the pooled proportion of recurrence. Subgroup analyses were conducted based on the reported individual recurrence in each surgical treatment category, data type, grade of epithelial dysplasia, and anatomical subsite of OL. The risks of recurrence between clinical subtypes, size of OL, surgical margin, and risk habits were compared through dichotomous meta-analysis using risk ratios under a random-effects model. The risk of MT with respect to the recurrence status of OL was also compared through risk ratio. Meta-regressions were performed to examine the association between mean age, male-to-female ratio, and reported follow-up period and recurrence. Heterogeneity among the studies was explored using *I*^2^ statistics. Sensitivity analysis was performed in high heterogeneity by removing influential studies sequentially. Since a dichotomous direct comparison meta-analysis with 2 similar treatments of OL was not possible with the available data, a network meta-analysis (NMA) was performed to compare the recurrence rates between various surgical treatments. Details of the study selection for NMA are presented in the Appendix Methods. Publication bias was analyzed by drawing funnel plots and Egger’s and Begg’s tests.

### Quality of Evidence

We used the GRADE rating system (Brennan and Johnston 2023) to evaluate the certainty of evidence for 2 of the main study outcomes, the proportion of OL recurrence after various surgical treatment modalities and the risk of MT based on OL recurrence. GRADEpro software was used to generate the quality of the evidence table (GRADEpro GDT 2024).

## Results

### Search Results

**Figure 1.**
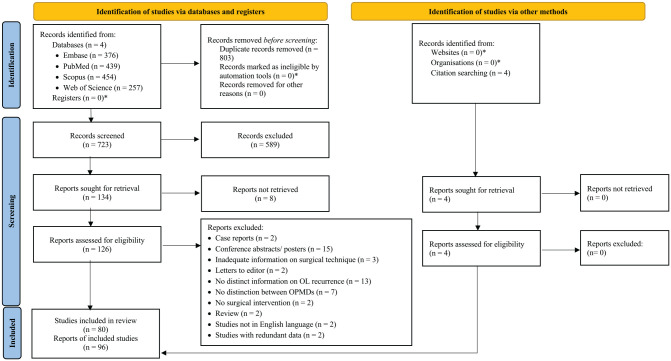
Preferred Reported Items for Systematic Reviews and Meta-Analyses flow diagram of study selection.

The initial search yielded 1,526 studies. After removing the duplicate records (*n* = 803), 723 studies remained. Of these, 589 were excluded after screening by title and abstract. Full texts were retrieved for the remaining 134 articles. In total, 130 studies could be screened by full text, of which 80 were selected for qualitative and quantitative analysis. The study selection process with exclusion at each step is presented in Figure 1.

### Study Characteristics

The general characteristics of the included studies are summarized in Appendix Table 1; the references to the studies are presented in Appendix References to included studies. In brief, the included studies were conducted in 21 countries between 1968 and 2024. Sixty-eight were observational studies (37 prospective and 31 retrospective), and 12 were randomized controlled trials (RCTs). Out of the 80 studies, 61 consisted of a single treatment modality (single-arm studies), while 19 included at least 2 types of interventions (multiarm studies). Data from each intervention from the multiarm studies were extracted and grouped with similar treatment modalities from single-intervention studies. Hence, 80 studies resulted in 96 single-intervention data sets. While CO_2_ laser (either excision and/or vaporization) was the most frequently reported surgical method (in 43 studies), Nd:YAG laser excision was the least reported technique (only in 2 studies). The number of OL lesions included in the individual studies ranged from 5 to 2,347, bringing the total number of samples up to 7,614 OL lesions. The male-to-female ratio was 1.65:1. The follow-up period ranged from 1 to 120 mo.

### RoB Assessment

The results for the risk of bias assessment are presented in Appendix Results and illustrated by Appendix Figures 1 and 2.

### Pooled Proportion of Recurrence of OL after Surgical Removal

Recurrence was defined as the reappearance of OL at the same site as a previously clinically completely excised lesion. The pooled proportion of OL recurrence for all treatment modalities was 22% (0.22; 95% confidence interval [CI], 0.18–0.25). The heterogeneity among the included studies was high (overall *I*^2^ = 93.13%). The forest plot for the pooled proportion of OL recurrence is shown in Appendix Figure 3. The assessment for publication bias for the pooled proportion of OL recurrence showed marked asymmetry in the funnel plot (Appendix Fig. 4). Furthermore, Egger’s and Begg’s tests for bias assessment showed significant small-study effects (Egger’s test, *P* < 0.001; Begg’s test, *P* = 0.0006).

### Subgroup Analysis Based on Surgical Modality, Data Type, Grades of Epithelial Dysplasia, and Anatomical Site

**Figure 2.**
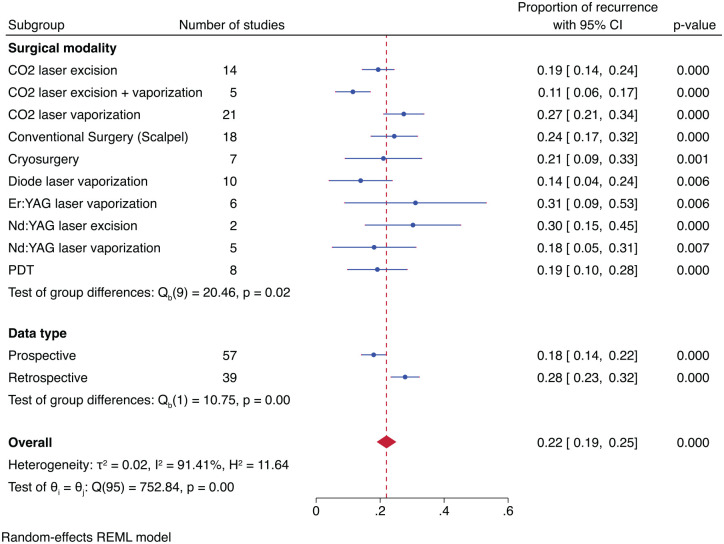
Forest plots illustrating recurrence rates with respect to surgical modality and type of data.

To examine individual proportions of recurrence related to surgical excision modalities, a subgroup meta-analysis (10 treatment groups) was performed. The proportions of recurrence identified for the surgical techniques, arranged from the most effective (least recurrence) to the least effective (most recurrence) modality (Fig. 2), were CO_2_ excision + vaporization (11%) > diode laser vaporization (14%) > Nd:YAG vaporization (18%) > CO_2_ laser excision (19%) > PDT (19%) > cryosurgery (21%) > conventional surgery (scalpel) (24%) > CO_2_ laser vaporization (27%) > Nd:YAG laser excision (30%) > Er:YAG laser vaporization (31%). The difference in proportions of recurrence across the treatment modalities was statistically significant (*P* = 0.02). The subgroup analysis based on data type produced a pooled proportion of recurrence of 28% for the retrospective and 18% for the prospective data, and the result was statistically significant (*P* = 0.001) (Fig. 2).

Next, a subgroup meta-analysis based on grades of epithelial dysplasia was performed. The estimated proportion of recurrence was highest in the severe dysplasia group (30.8%), followed by no dysplasia (25.7%), moderate dysplasia (24.5%), and mild dysplasia (24.3%). However, the difference in proportions of recurrence across the dysplasia grades was not statistically significant (*P* = 0.78). The overall heterogeneity across all subgroups was high, *I*^2^ = 71.79%.

**Figure 3.**
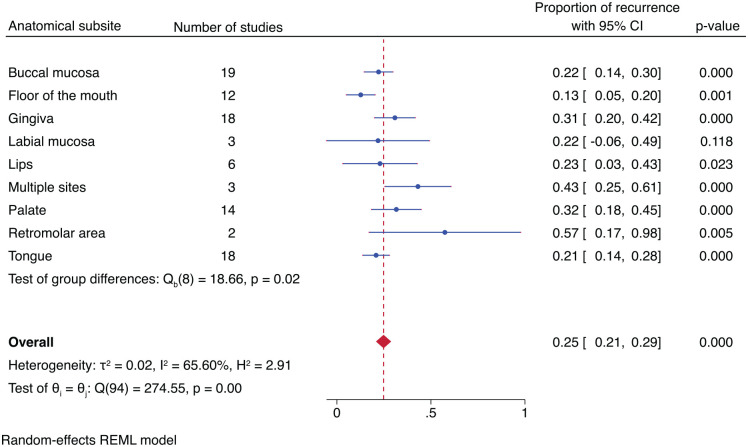
Forest plot showing proportions of recurrence of oral leukoplakia (OL) based on anatomical sites. OL in the retromolar area, multiple sites, and palate had higher proportions of recurrence.

The subgroup analysis for the proportion of recurrence based on anatomical subsites showed a higher recurrence for OL in the retromolar area (57%), OL distributed in multiple sites (43%), palate (32%), and gingiva (31%) than in floor of the mouth (13%), tongue (21%), and buccal mucosa (25%). The recurrence proportion was statistically significant across the anatomical subsites with moderate heterogeneity (*P* = 0.02; *I*^2^ = 65.6%) (Fig. 3).

### Recurrence Risk Based on Clinical Subtypes, Surgical Margin, Size, and Risk Habits

**Figure 4.**
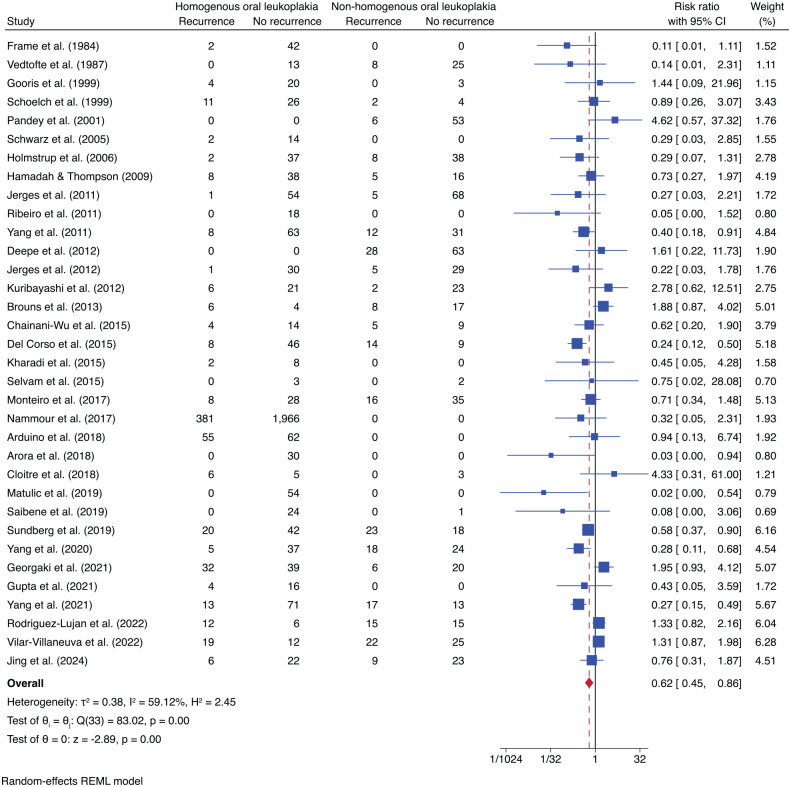
Forest plot illustrating the risk of recurrence between homogeneous oral leukoplakia (HOL) and nonhomogeneous oral leukoplakia (NHOL). The risk ratio of oral leukoplakia recurrence between HOL and NHOL is 0.62, indicating that HOL has 38% less risk of recurrence than NHOL.

Thirty-four studies reported recurrence based on the clinical subtypes of OL (i.e., HOL and NHOL). HOL lesions were found to have 38% less risk of recurrence than NHOL lesions (risk ratio [RR] = 0.62; 95% CI, 0.4–0.86; *P* = 0.04). The included studies had moderate heterogeneity (*I*^2^ = 59.12%) (Fig. 4).

Four studies reported OL recurrence based on the surgical margin. OL excised with no/inadequate surgical margin had a significantly higher risk of recurrence than those removed with clinically evaluated adequate surgical margin (RR = 2.96; 95% CI, 1.31–6.71; *P* = 0.01). This outcome had a low heterogeneity (*I*^2^ = 46.17%) (Appendix Fig. 5).

The meta-analysis results showed no significant differences in the risk of OL recurrence between larger and smaller lesions (Appendix Fig. 6A, B). Similarly, among smoking, alcohol, and betel quid chewing habits (current versus past/no habit), only the betel quid chewing habit had a significant risk associated with OL recurrence (Appendix Fig. 7).

### Meta-regression with Other Clinical Variables

**Figure 5.**
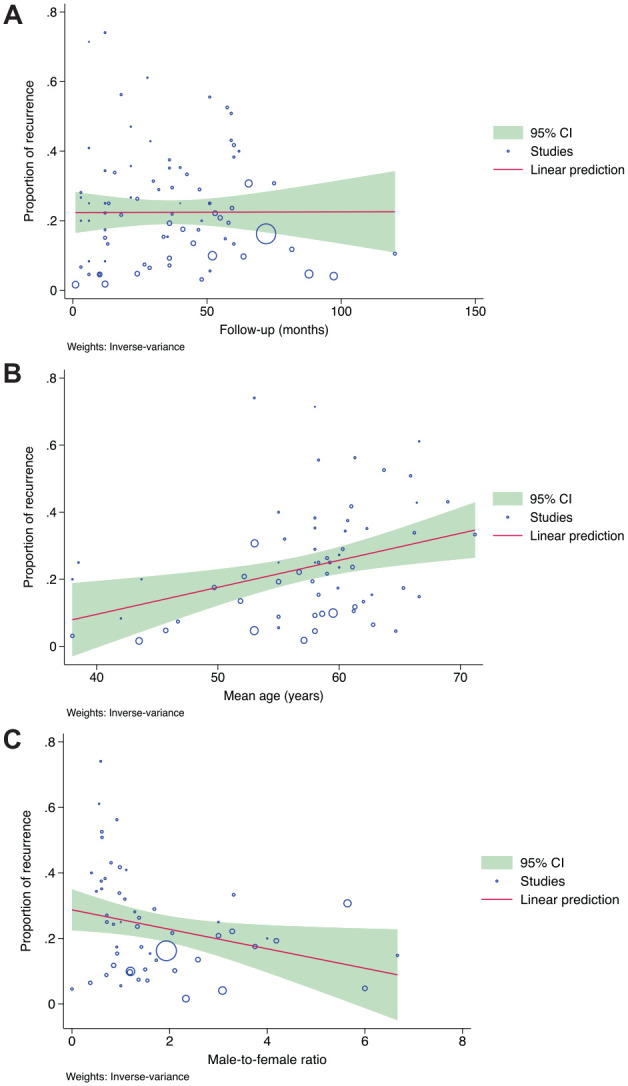
(**A**) Meta-regression analysis showed a flat regression line, indicating a nonlinear relationship and no association with the follow-up period. (**B**) Mean age (years) showed a linear and positive correlation, indicating that the recurrence rate of OL is higher in patients with advanced age. (**C**) The male-to-female ratio showed a linear and negative correlation, indicating that females have a higher propensity for recurrence than males.

Meta-regression with 3 covariates, follow-up period, mean age of the patients, and the male-to-female ratio, was performed to analyze the association between these variables with the proportion of OL recurrence. The follow-up period (in months) was not associated with the recurrence rate (coefficient = 0.000, *P* = 0.979). While the mean age (in years) was significantly positively associated with the recurrence rate (coefficient = 0.008, *P* = 0.003), the male-to-female ratio had a significant negative association with the recurrence rate (coefficient = −0.029, *P* = 0.03) (Fig. 5).

### MT Risk Based on the Recurrence Status of OL

**Figure 6.**
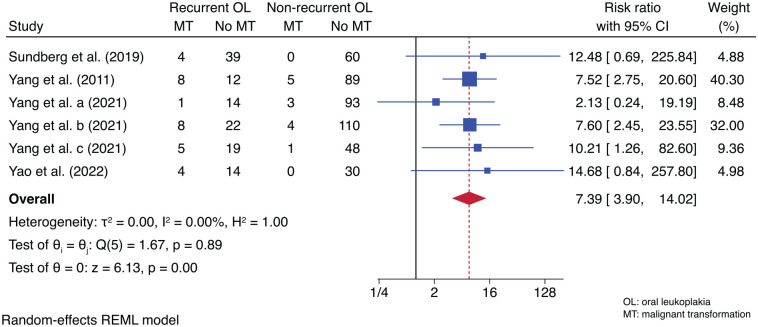
Forest plot for the risk of malignant transformation between recurrent and nonrecurrent oral leukoplakia (OL). Recurrent OL had a 7.39 times higher risk for malignant transformation compared to nonrecurrent OL.

Six studies reported MT based on the recurrence status of OL. The meta-analysis result showed a 7.39 times higher risk of MT for recurrent OL compared to the nonrecurrent lesions (RR = 7.39; 95% CI, 3.90–14.02; *P* < 0.001). This outcome had no heterogeneity (*I*^2^ = 0.00%) (Fig. 6).

### NMA Comparing Multiple Treatment Groups

The results of the NMA are presented in Appendix Results and depicted in Appendix Figure 8.

### Quality of Evidence

As per the GRADE criteria, the evidence from the studies that provided information on the proportion of OL recurrence after various surgical treatments was low, except for Nd:YAG laser excision, which was moderate (Appendix Table 2). As for the risk of MT based on the recurrence status of OL, the evidence was high, mainly because of a very strong association between MT and the recurrence status of OL (Appendix Table 3).

## Discussion

The current study represents one of the most extensive meta-analyses conducted on the recurrence rate of OL following 10 different surgical treatments. From 80 studies included in the meta-analysis, a pooled proportion of recurrence of 22% was observed in our study. To the best of our knowledge, this is the first study that has reported the pooled and individual recurrence proportions of OL after various surgical treatments.

The proportion of OL recurrence after laser-based treatments ranged from 11% to 31% in our study. This finding aligns with the pooled recurrence rate of 16.5% reported in the meta-analysis by de Pauli Paglioni et al. (2020). However, the present study reported OL recurrence rates after specific laser treatments in terms of the laser source (e.g., CO_2_, diode, Er:YAG, Nd:YAG) and the application techniques (excision, vaporization, or a combination of excision and vaporization). We found that the CO_2_ laser, irrespective of the application techniques, had excellent outcomes regarding OL recurrence. Our finding is in line with the conclusions drawn in the systematic review by Mogedas-Vegara et al. (2016), where CO_2_ lasers were reported to be the safest, least morbid, and most effective in treating OL because of their precise and controlled tissue damage, complete depth control, and sound wound-healing effects. Of note, the combination of CO_2_ laser excision and vaporization showed lower recurrence than CO_2_ laser alone or CO_2_ laser vaporization alone. This finding could be related to a thorough depth and width control of tissue achieved through excision and vaporization, possibly leaving the least residual tissue behind.

Interestingly, the current study found a recurrence proportion of 14% for OL treated with diode laser vaporization. This observation might be related to the ability of high-power diode lasers to cause deeper thermal damage to tissues compared to CO_2_ lasers (Gupta et al. 2021). However, Er:YAG laser vaporization and Nd:YAG laser excision alone had higher recurrence rates in our results. The higher recurrence rate for laser vaporization alone could be attributed to the limited tissue penetration ability, which might not be adequate for the ablation of atypical/dysplastic epithelial cells (Del Corso et al. 2015; Petrov et al. 2021). Moreover, the recurrence rate of OL after Nd:YAG laser excision in the present study should be viewed in the context that the result came from only 2 studies.

In the present study, conventional scalpel excision and cryosurgery showed 24% and 21% recurrence rates, respectively, aligning with reports from previous studies (Sako et al. 1972; Prasad et al. 2009; Kawczyk-Krupka et al. 2012; Monteiro et al. 2017; Sundberg et al. 2019). Both methods are considered less effective in terms of margin control, postoperative discomfort, and fibrotic scarring compared to laser excision (Ishii et al. 2004).

Besides the choice of excision, several clinical and histopathological factors of OL are linked to the risk of OL recurrence (Napier and Speight 2008; Balasundaram et al. 2014). In the current meta-analysis, NHOL, advanced age, female sex, poor or inadequate surgical margin, specific anatomical subsites (retromolar area, multiple sites, palate, and gingiva), and retrospective data showed a significantly higher association with recurrence of OL.

Similar to our findings, NHOL has been reported as a risk factor for recurrence and MT of OL (Warnakulasuriya and Ariyawardana 2016; Sundberg et al. 2019; Paglioni et al. 2022). However, some studies have reported that the risk of OL recurrence is independent of the clinical presentation of the lesion (Galletta et al. 2017; Rodriguez-Lujan et al. 2022). Interestingly, in contrast to previous reports (Yang, Lee, Chang, et al. 2021; Rodriguez-Lujan et al. 2022), we found a significant positive association between OL recurrence and advanced age. Our results are in line with previous studies where advanced age has been reported to be associated with MT of OL (Warnakulasuriya et al. 2011; Liu et al. 2012; Paglioni et al. 2022). Our finding that female sex has a higher propensity for OL recurrence and MT aligns with previous reports (Napier and Speight 2008; Warnakulasuriya and Ariyawardana 2016; Lorini et al. 2022). However, several studies have reported a lack of association between sex and OL recurrence or MT (Galletta et al. 2017; Sundberg et al. 2019; Yang, Lee, Wu, et al. 2021).

We found that OL excised with inadequate surgical margin has a higher risk of recurrence, which parallels with conclusions from previous studies (Kuribayashi et al. 2012; Chainani-Wu et al. 2015). From a surgical standpoint, it seems reasonable that inadequate surgical margins leave residual tissue behind, which could propagate and lead to relapse.

We found a statistically higher reported recurrence in retrospective data than in longitudinal data. Although patient and lesion-related factors could be related to this observation, the influence of the risk of overestimation of recurrences in retrospectively collected data cannot be ruled out. Indeed, unlike prospective data collection, it is difficult to precisely differentiate a recurrence from a second primary lesion in retrospective data unless the lesions are appropriately documented with clinical pictures.

In corroboration with previous studies (Kuribayashi et al. 2012; Sundberg et al. 2019; Rodriguez-Lujan et al. 2022), there was no significant difference between the grades of epithelial dysplasia and OL recurrence in the present study. It is probable that after surgical removal, lesions with higher dysplasia grades may undergo MT and, therefore, be lost to follow-up. Furthermore, categorizing epithelial dysplasia is subjective and has significant intra- and interobserver variability (Kujan et al. 2007). This might have influenced the results drawn for OL recurrence based on dysplasia grades across the studies.

A recurrence is more likely to occur and subsequently be detected if the patient is followed up for a long period in contrast to a short period and will be missed and thereby underreported if the follow-up is shorter (Pindborg et al. 1968). In contrast to a previous report (Sundberg et al. 2019), increased follow-up periods were not related to a higher OL recurrence in the present study. This observation could be related to the fact that both prospective and retrospective studies with different follow-up periods were included in the present meta-analysis and that retrospective data collection is often associated with selection bias. Hence, it is advisable that studies should report recurrence per follow-up year.

We found a 7.39 times higher risk of MT for recurrent OL compared to nonrecurrent OL. Previous studies have also reported recurrence status as an independent risk factor for MT (Yang et al. 2010, Yao et al. 2022). This implies that proper management and follow-up of recurrent OL could be key to minimize MT. Thus, it is worthwhile that future studies report the MT of OL based on its recurrence. Furthermore, molecular studies might elucidate the precise mechanisms of how recurrent OL poses an increased risk of MT compared to nonrecurrent OL.

The findings of the NMA are discussed in Appendix Discussion.

### Strengths and Limitations of the Study

This systematic review and meta-analysis, including NMA, is the first to report a pooled recurrence of OL after conventional scalpel excision, different types of laser therapies, cryotherapy, and PDT. Our results are based on a large sample size of OL subjected to various treatment modalities. Furthermore, the study included analyses of the interaction between clinical factors that influence OL recurrence.

However, there are some limitations of this study. First, the meta-analysis results were based on the assumption that the baseline population characteristics were evenly distributed across the studies involved. Second, despite being able to deduce recurrence rates after individual treatment as single-arm interventions and compare them indirectly, a head-to-head comparison between the treatments was not possible, mainly due to a lack of comparative studies for such analysis. Similarly, we could not examine the association between recurrence and histopathological status of surgical margins and the depth of the lesion because of limited data availability.

Third, the meta-analysis results showed moderate to high heterogeneity, which remained largely unchanged even after removing studies with unclear methodology or ambiguous reporting of recurrence and clinical parameters. Furthermore, we could not observe statistically significant changes in the overall effect sizes in our sensitivity analysis, suggesting that our estimates were stable and robust. The heterogeneity in our results might stem from the diverse ways of using lasers or different substrates for PDT, which can influence the recurrence. Another source of heterogeneity could be related to the way recurrence was interpreted and reported in the included studies. However, strict inclusion criteria were used to define recurrence in the current study. Finally, there was significant publication bias among the studies included in the meta-analysis which could influence the current findings

## Conclusion

The results of the present study revealed that laser-based surgeries might decrease the recurrence rate of OL compared to other surgical approaches. Female sex, advanced age, nonhomogeneous OL, and OL with inadequate surgical margins have a greater propensity for recurrence. Therefore, OL patients with such clinicopathological features should be considered for a close follow-up regimen after surgical therapy. Furthermore, recurrent OL needs to be approached more comprehensively since it possesses a greater risk of MT. Since the contemporary evidence on determinants of OL recurrence is obtained primarily from single-arm studies, prospective studies are necessary to compare various surgical techniques and identify the clinicopathological determinants of recurrence.

## Author Contributions

B.P. Bhattarai, A.K. Singh, contributed to conception, design, data acquisition, analysis, and interpretation, drafted and critically revised the manuscript; R.P. Singh, contributed to data interpretation, critically revised the manuscript; R. Chaulagain, contributed to data analysis and interpretation, critically revised the manuscript; T.M. Søland, contributed to data interpretation, drafted and critically revised the manuscript; B. Hasséus, contributed to conception, data interpretation, drafted and critically revised the manuscript; D. Sapkota, contributed to conception, design, data analysis and interpretation, drafted and critically revised the manuscript. All authors have their final approval and agree to be accountable for all aspects of work.

## Supplemental Material

sj-docx-1-jdr-10.1177_00220345241266519 – Supplemental material for Recurrence in Oral Leukoplakia: A Systematic Review and Meta-analysis Supplemental material, sj-docx-1-jdr-10.1177_00220345241266519 for Recurrence in Oral Leukoplakia: A Systematic Review and Meta-analysis by B.P. Bhattarai, A.K. Singh, R.P. Singh, R. Chaulagain, T.M. Søland, B. Hasséus and D. Sapkota in Journal of Dental Research
