# Supplementary material for: Recurrence in Oral Leukoplakia: A Systematic Review and Meta-analysis
Source: J Dent Res. 2024 Sep 18;103(11):1066–75. doi: 10.1177/00220345241266519 (PMC11504345; doi:10.1177/00220345241266519)
Supplement: sj-docx-1-jdr-10.1177_00220345241266519 – Supplemental material for Recurrence in Oral Leukoplakia: A Systematic Review and Meta-analysis [file sj-docx-1-jdr-10.1177_00220345241266519.docx]

**Recurrence in oral leukoplakia: a systematic review and meta-analysis.**

Bishwa Prakash Bhattarai, Ashutosh Kumar Singh, Rabindra Pratap Singh, Rajib Chaulagain, Tine Merete Søland, Bengt Hasséus, Dipak Sapkota

**Appendix**

**Methods**

***Search strategies and electronic databases consulted***

**EMBASE search strategy**

Search: Database inception to April 2024

(oral and leukoplakia and recurrence).af.

**PubMed search strategy**

Search: Database inception to April 2024

**Query**

Search: ((oral) AND (leukoplakia)) AND (recurrence)

("mouth"[MeSH Terms] OR "mouth"[All Fields] OR "oral"[All Fields]) AND ("leucoplakias"[All Fields] OR "leukoplakia"[MeSH Terms] OR "leukoplakia"[All Fields] OR "leucoplakia"[All Fields] OR "leukoplakias"[All Fields]) AND ("recurrance"[All Fields] OR "recurrence"[MeSH Terms] OR "recurrence"[All Fields] OR "recurrences"[All Fields] OR "recurrencies"[All Fields] OR "recurrency"[All Fields] OR "recurrent"[All Fields] OR "recurrently"[All Fields] OR "recurrents"[All Fields])

**Translations**

**oral:** "mouth"[MeSH Terms] OR "mouth"[All Fields] OR "oral"[All Fields]

**leukoplakia:** "leucoplakias"[All Fields] OR "leukoplakia"[MeSH Terms] OR "leukoplakia"[All Fields] OR "leucoplakia"[All Fields] OR "leukoplakias"[All Fields]

**recurrence:** "recurrance"[All Fields] OR "recurrence"[MeSH Terms] OR "recurrence"[All Fields] OR "recurrences"[All Fields] OR "recurrencies"[All Fields] OR "recurrency"[All Fields] OR "recurrent"[All Fields] OR "recurrently"[All Fields] OR "recurrents"[All Fields]

**Scopus search strategy**

Search: Database inception to April 2024

**Query**

( TITLE-ABS-KEY ( oral ) AND TITLE-ABS-KEY ( leukoplakia ) AND TITLE-ABS-KEY ( recurrence ) )

**Web of Science search strategy**

Search: Database inception to April 2024

**Query**

((ALL=(oral)) AND ALL=(leukoplakia)) AND ALL=(recurrence)

***Details of the study selection for Network meta-analysis (NMA)***

Eleven studies (Arduino et al. 2018; Del Corso et al. 2015; Georgaki et al. 2021; Ishii et al. 2004; Kawczyk-Krupka et al. 2012; Monteiro et al. 2017; Natekar et al. 2017; Petrov et al. 2021; Sai Praveen et al. 2021; White et al. 1998; Yasmeen et al. 2019) that qualified for the network meta-analysis had reported OL recurrence between two or more treatment modalities. Eight of the eleven studies were two-arm, and three (Ishii et al. 2004; Monteiro et al. 2017; Natekar et al. 2017) were multi-arm. There was a total of 769 patients.

**Results**

***Risk of Bias (RoB) Assessment***

Among the 68 observational studies, six had high RoB (“X”), one had low RoB (“+”), and the remaining 61 had moderate RoB (“-”), as illustrated in Appendix Figure 1. Among the individual domains across the studies, “*adequate participation rate*” had the lowest bias, whereas “*sample size and power estimation”* had the highest risk of bias. Regarding the RoB assessment for twelve RCTs included in the meta-analysis, two had low RoB, and ten had moderate RoB (Appendix Figure 2).

***Network meta-analysis (NMA) comparing multiple treatment groups***

We performed the NMA using the frequentist approach. The treatment modalities were grouped into five arms: laser excision, laser vaporization, conventional scalpel surgery, cryosurgery, and PDT. The network was connected, making indirect comparisons between the treatment modalities possible (Appendix Figure 8A). The total number of events (recurrence of OL) in the network was 225. The forest plot (Appendix Figure 8B) illustrates the risk difference in OL recurrence between other treatment modalities compared to conventional scalpel surgery as the reference standard. Based on the risk of OL recurrence, the treatments could be ranked from the best to the worst as laser excision > laser vaporization > conventional scalpel surgery > cryotherapy > PDT.

**Discussion**

***Findings from the network meta-analysis (NMA)***

The findings of the NMA, by and large, paralleled the results of the meta-analysis. From the NMA results, laser excision stood out as the most effective treatment with the least risk of recurrence, followed by laser vaporization and scalpel excision. However, cryosurgery and PDT, with better outcomes than conventional scalpel excision in the meta-analysis, had poorer results in the NMA. These results could be due to the low number of comparative studies on cryosurgery, or PDT, included in the NMA, highlighting the need for more comparative studies or RCTs.

**Appendix Figure 1:** Distribution of risk of bias within individual observational studies (A). Overall risk of bias in each domain across the observational studies (B).

**Appendix Figure 2:** Risk of bias within individual RCTs (A) and in each domain across the RCTs (B).

**Appendix Figure 3:** Forest plot illustrating the pooled proportion of OL recurrence after various surgical treatments from eighty studies; the recurrence proportion is 22%.

**Appendix Figure 4:** Significant asymmetry can be observed on the funnel plot, indicating the presence of a publication bias.

**Appendix Figure 5:** Forest plot for the risk of recurrence between OL with no/ inadequate- and adequate surgical margin. OL with no/inadequate surgical margins had a 2.96 greater risk of recurrence than those with adequate margins.

**Appendix Figure 6A:** Forest plot for the risk of recurrence between OL smaller and larger than 2 cm. No significant difference in risk of recurrence is seen.

**Appendix Figure 6B:** Forest plot for the risk of recurrence between OL smaller and larger than 200 mm^2^. The risk ratio of 0.48, CI: 0.20 to 1.12, suggested no significant difference between the smaller and the larger lesions for OL recurrence.

**Appendix Figure 7:** Forest plot for risk of OL recurrence between current habits and past/no habits. The risk of OL recurrence was associated only with betel quid chewing habits (Risk ratio = 2.17; CI= 1.07 to 4.42 p= 0.03).

**Appendix Figure 8:** Plots showing results from the network meta-analysis. Network of included studies with respect to five treatment modalities (A). The thickness of the line and numbers represent the number of studies included in each comparison. Forest plot showing the overall comparison of different treatment modalities with conventional scalpel surgery (B). Laser excision was associated with the least risk for OL recurrence compared to other treatment modalities.

**Appendix Table 1:** General characteristics of included studies

| **Author** | **Country** | **Study type** | **Surgical modality** | **Total sample** | **M** | **F** | **Age (Mean / Range) (years)** | **Follow-up duration (months)** | **Anatomical site** | **Recurrence** | **HOL/ NHOL** | **MT; New Lesions** | **DFS** |
| --- | --- | --- | --- | --- | --- | --- | --- | --- | --- | --- | --- | --- | --- |
| Pindborg et al. (1968) | Denmark | Prospective | Conventional scalpel surgery | 21 | - | - | - | 3- 108 | - | 2 | - | - | - |
| Sako et al.  (1972) | USA | Prospective | Cryosurgery | 60 | - | - | - | (30- 54) | - | 12 | - | MT- 4/60 | - |
| Horch et al.  (1982) | Germany | Prospective | CO2 laser vaporization | 30 | - | - | - | 13 | T, BM, G, L, AM, FoM | 4 | - | - | - |
| Frame et al.  (1984) | UK | Prospective | CO2 laser surgery + CO2 vaporization | 44 | 15 | 19 | 58 | 10 (4- 24) | FoM, VT, BM, DT, LT, LM, L, AM, HP, SP | 2 | 44/0 | MT- 0/44 | - |
| Horch et al.  (1984) | Germany | Prospective | CO2 laser vaporization | 32 | - | - | - | 37 | T, BM, G, L, AM, FoM | 7 | - | - | - |
| Silverman et al.  (1984) | USA | Prospective | Conventional scalpel surgery | 61 | - | - | - | - | - | 21 | - | - | - |
| Vedtofte et al  (1987). | Denmark | Prospective | Conventional scalpel surgery | 46 | 27 | 19 | 65.3 | 46.6 | BM, AM, LM, FoM, T, P | 8 | 13/33 | MT- 3/46 | - |
| Chu et al.  (1988) | USA | Retrospective | CO2 laser excision | 34 | 12 | 17 | 55 | (36-114) | BM, FoM, G, LT, DT, VT, SP | 3 | - | MT- 1/38 | - |
| Cheisa et al.  (1990) | Italy | Retrospective | CO2 laser excision | 140 | 113 | 27 | 55±10.5 | 36 | L, T, FoM, BM, G, P | 27 | 36/104 | MT- 2/140, New lesions- 29/140 | 3 years- 0.57 (0.47 to 0.67) |
| Roodenburg et al.  (1991) | Netherlands | Retrospective | CO2 laser vaporization | 103 | 38 | 32 | 58.6 | 63.6 | L, BM, G, FoM, P, T, FP | 10 | 62/41 | MT- 0 | - |
| Cheisa et al.  (1993) | Italy | Retrospective | CO2 laser excision | 167 | 128 | 39 | 56.7±9.7 | 53±36 | L, T, FoM, BM, G, P | 37 | 52/115 | MT- 6/167, New lesions- 38/167 | 3 years- 70.7%; 5years- 51.5% |
| White et al.  (1998) | USA | Prospective | CO2 laser excision + vaporization | 17 | - | - | 60±17 | - | T, G, BM, LM, L, HP, SP, FoM | 4 | - | - | - |
|  |  |  | Nd:YAG excision | 22 | - | - |  | - |  | 6 |  |  |  |
| Schoelch et al.  (1999) | USA | Prospective | *CO2 & Nd:YAG Laser excision* | 43 | 32 | 24 | 63 | 32 (6- 178) | G, BM, FoM, L, P, LM, | 13 | 37/6 | MT- 3/43 | - |
| Gooris et al.  (1999) | Netherlands | Retrospective | CO2 laser vaporization | 27 | 20 | 3 | 66.6 | 75.6 (12-192) | Lower lip | 4 | 24/3 | MT- 0; New lesions- 4/27 | - |
| Pandey et al.  (2001) | India | Prospective | Conventional scalpel surgery | 59 | 40 | 19 | - | 12- 37 | BM, T, LM | 6 | 0/59 | MT- 0/59 | 3 years- 0.62 (95% CI: 0.36- 0.87) |
| Thomson & Wylie  (2002) | UK | Retrospective | CO2 laser excision | 62 | 37 | 20 | 59 | 18 (1- 44) | FoM, VT, LT, BM, RA, P, AM | 19 | 43/19 | - | - |
| Sieron et al.  (2003) | Poland | Prospective | PDT | 24 | - | - | - | 4- 34 | BM, G, AM, L | 1 | - | - | - |
| Deepe et al.  (2004) | Germany | Prospective | CO2 laser vaporization | 61 | - | - | - | 37 | - | 18 | 21/40 | - | - |
| Ishii et al.  (2004) | Japan | Retrospective | Cryosurgery | 7 | - | - | 58 | 6 | G, T, BM, P, FoM, L | 5 | - | MT- 0/7 | - |
|  |  |  | Conventional scalpel surgery | 8 |  |  |  |  |  | 2 |  | MT- 3/8 |  |
|  |  |  | Laser Excision | 9 |  |  |  |  |  | 2 |  | MT- 0/9 |  |
|  |  |  | Laser Vaporization | 73 |  |  |  |  |  | 22 |  | MT- 1/73 |  |
| Chandu & Smith  (2005) | Australia | Retrospective | CO2 vaporization | 69 | 27 | 16 | 60.3±13.6 | 47.2±28.2 | - | 20 | - | MT-5/69 | 3 years- 55.4% (95% CI: 38.3- 72.5); 5 years- 33.9% (95% CI: 10.2- 57.5) |
| van der Hem et al.  (2005) | Netherlands | Prospective | CO2 laser vaporization | 282 | 109 | 91 | 59.5 | 52 (1-219) | L, LM, BM, T, P, G, FP | 28 | 136/61 | MT- 3/282 | - |
| Schwarz et al.  (2005) | Germany | RCT | CO2 Laser vaporization | 8 | 5 | 5 | 55±12 | 51.1±25.4 | LM, FoM, G, LT | 2 | 8/0 | - | - |
|  |  |  | Er:YAG Laser vaporization | 8 | 5 | 5 |  |  |  | 0 | 8/0 |  |  |
| Holmstrup et al.  (2006) | Denmark | Retrospective | Conventional scalpel surgery | 85 | 41 | 48 | 61.3 | 81.6 (18- 223.2) | FoM, VT, LT, DT, BM, P, G, L | 10 | 39/46 | MT-11/85 | - |
| Vivek et al.  (2008) | India | Prospective | Nd:YAG laser vaporization | 28 | 17 | 11 | - | 36 | LM, BM, G, T | 2 | - | MT-3/28 | - |
| Hamadah & Thomson  (2009) | UK | Prospective | CO2 laser excision | 67 | 51 | 27 | 57.8 | 58±27 | FoM, VT, BM, SP, G | 13 | 46/21 | MT- 3/67 | 2 years- 86%; 5years- 69% |
| Prasad et al.  (2009) | India | Retrospective | Cryosurgery | 20 | 15 | 5 | 38.5 | 12 | - | 5 | - | - | - |
| Yang et al.  (2011) | Taiwan | Retrospective | CO2 laser excision | 114 | 90 | 24 | 49.7±12.2 | 41±16 | L, BM, G, FoM, P, T, RA | 20 | 71/43 | MT- 13/114 | - |
| Chiu et al.  (2011) | Taiwan | Retrospective | Conventional scalpel surgery | 73 | - | - | - | 24-48 | - | 31 | - | - | - |
| Jerges et al.  (2011) | UK | Prospective | PDT (ALA or mTHPC) | 128 | 82 | 65 | 53±8.9 | 88 | FoM, LT, DT, VT, BM, HP, AM, RA, Tuberosity | 6 | 55/73 | MT- 11/147 | - |
| Reibero et al.  (2011) | Brazil | RCT | Cryosurgery | 10 | 0 | 10 | 64.67±11.31 | 9.9 | BM, AM, T, P, G | 0 | - | - | - |
|  |  |  | *Cryosurgery + GaAlAs laser therapy* | 8 | 3 | 5 | 64.67±11.31 | 8.25 |  | 0 | - | - | - |
| Jerges et al.  (2012) | UK | Prospective | CO2 laser excision + vaporization | 65 | 47 | 30 | 58±4.8 | 36 | FoM, LT, DT, VT, BM | 6 | 31/34 | MT- 8/77 | - |
| Deepe et al.  (2012) | Germany | Prospective | CO2 laser vaporization | 91 | - | - | - | 75 (4- 123) | - | 28 | 0/91 | MT-1/120 | - |
| Kuribayashi et al.  (2012) | Japan | Retrospective | Conventional scalpel surgery | 44 | 25 | 27 | 58.3 | 33.8±18.4 | G, T, P, BM | 8 | 21/23 | MT- 1/44 | - |
| Montebugnoli et al.  (2012) | Italy | Prospective | Nd:YAG laser vaporization | 13 | 8 | 5 | 62.7±9.9 | 35.3±19.1 | G, P, T | 2 | - | - | - |
| Kawczyk-Krupka et al.  (2012) | Poland | Prospective | Cryotherapy | 37 | 17 | 20 | 21- 73 | 2- 127 | BM, AR, L, FoM, P, T | 9 | - | - | - |
|  |  |  | PDT (ALA) | 48 | 20 | 28 | 32- 75 | 4- 34 |  | 13 |  |  |  |
| Thomas et al.  (2012) | India | Prospective | Conventional scalpel surgery | 98 | 74 | 24 | - | 97.20 | BM, T, L | 4 | 23/75 | MT-4/98; New lesion-15/98 | - |
| Brouns et al.  (2013) | Netherlands | Retrospective | CO2 laser vaporization | 35 | 10 | 25 | 55 | 61.9 (12- 179) | T, FoM, L, HP, BM, AM, G | 14 | 10/25 | MT- 5/35 | - |
| Vlk & Smucler  (2013) | Czech Republic | Prospective | Diode laser vaporization | 76 | 34 | 36 | 59 | 24 | T, BM, RA, L, P, AM | 20 | 44/32 | MT- 2/76 | - |
| Matsumoto et al.  (2015) | Japan | Prospective | CO2 laser surgery + CO2 vaporization | 38 | 21 | 14 | 61.5 | 120 | LT, DT | 4 | 23/15 | MT-0 |  |
| Chainani-Wu et al.  (2015) | USA | Retrospective | CO2 laser vaporization | 32 | 15 | 11 | - | 3 | BM, LT, VT, G | 9 | 18/14 | - | - |
| Mogedas-Vegara et al.  (2015) | Spain | Retrospective | CO2 laser vaporization | 65 | 32 | 33 | 66.2±13.1 | 15±10.6 | T, G, L, BM, FoM, RA, P | 22 | - | MT- 10/65 | - |
| Kharadi et al.  (2015) | India | Prospective | Diode laser vaporization | 10 | 8 | 2 | 43.7 | 6 | BM, LM | 2 | 10/0 | - | - |
| Kundoor et al.  (2015) | India | Prospective | Diode laser vaporization | 5 | - | - | - | 6 | - | 0 | - | - | - |
| Das et al.  (2015) | India | Prospective | Nd:YAG laser vaporization | 42 | 36 | 6 | 45.7 | 24 | BM, LT, G, P, LM | 2 | - | - | - |
| Selvam et al.  (2015) | India | Prospective | PDT (ALA) | 5 | 5 | 0 | 42±5.6 | 12 | G, BM, VT | 0 | 3/2 | - | - |
| Del Corso et al.  (2015) | Italy | Retrospective | Nd:YAG vaporization | 47 | 19 | 28 | 58 | 60±32.49 | T, FoM, HP, BM, G | 18 | 33/14 | MT- 3/47 | - |
|  |  |  | CO2 Laser excision | 30 | 19 | 11 | 62 | 60±32.49 |  | 4 | 21/9 | Mt- 0/30 |  |
| Galletta et al.  (2017) | Brazil | Prospective | *CO2 & Diode laser excision* | 40 | 23 | 17 | 60.5 | 22 (6- 71) | LT, BM, FoM, HP, G, AM, SP, DT, VT, RA | 11 | 32/17 | MT- 2/40; New lesion- 4/40 | - |
| Nammour et al.  (2017) | Belgium | Prospective | CO2 laser surgery + CO2 vaporization | 2347 | 845 | 437 | 40- 82 | 72 | T, AM, P, FoM, G | 381 | 2347/0 | MT-12/2347 | - |
| Monteiro et al.  (2017) | Italy | Retrospective | Conventional scalpel surgery | 17 | 35 | 52 | 59.4±13.9 | 21.6 (1- 151) | LM, FoM, T, BM, HP, G | 8 | 36/51 | - | - |
|  |  |  | *QMR Lancet* | 8 |  |  |  |  |  | 2 |  |  |  |
|  |  |  | *Er:YAG laser excision/vaporization* | 33 |  |  |  |  |  | 5 |  |  |  |
|  |  |  | Nd:YAG laser excision | 14 |  |  |  |  |  | 5 |  |  |  |
|  |  |  | CO2 laser vaporization | 15 |  |  |  |  |  | 4 |  |  |  |
| Natekar et al.  (2017) | India | RCT | Diode laser vaporization | 10 | 20 | 10 | 36 | 6 | - | 0 | - | - | - |
|  |  |  | CO2 laser vaporization | 10 |  |  |  |  |  | 0 |  |  |  |
|  |  |  | Cryosurgery | 10 |  |  |  |  |  | 0 |  |  |  |
| Cloitre et al.  (2018) | France | Retrospective | CO2 laser vaporization | 14 | 14 | 11 | 66.4±11.1 | 28.9 | T, L, AR, G, BM, HP | 6 | 11/3 | MT- 0/14 | - |
| Arora et al.  (2018) | India | Prospective | Diode laser vaporization | 30 | 21 | 9 | 43.5±10 | 1 | BM, LM, T | 0 | 30/0 | - | - |
| Arduino et al.  (2018) | Italy | RCT | Conventional scalpel surgery | 58 | 20 | 25 | 68.96±13.41 | 59±17.2 | G, P, BM, T | 25 | 58/0 | - | - |
|  |  |  | Er:YAG laser vaporization | 59 | 16 | 26 | 65.89±11.91 | 59±17.2 |  | 30 | 59/0 |  |  |
| Saibene et al.  (2019) | Italy | Retrospective | CO2 laser excision | 25 | - | - | 56.7±15.55 | - | T, BM, HP, G, L, SP, RA, FoM | 0 | 24/1 | - | - |
| Sundberg et al.  (2019) | Sweden | Prospective | Conventional scalpel surgery | 103 | 51 | 52 | 61 | 60 | FoM, BM, LT, VT, DT, SP, HP, AM, G, L | 43 | 62/41 | - | - |
| Gabric et al.  (2019) | Croatia | Prospective | Er:YAG laser vaporization | 27 | 10 | 17 | 53±13 | 12 | BM, T, FoM | 20 | - | - | - |
| Matulic et al.  (2019) | Croatia | RCT | Er:YAG laser vaporization | 27 | 16 | 38 | 57.1 | 12 | BM, FoM | 0 | 27/0 | - | - |
|  |  |  | *Er,Cr:YSGG laser vaporization* | 27 |  |  |  |  |  |  | 27/0 |  |  |
| Yasmeen et al.  (2019) | India | RCT | Diode laser vaporization | 15 | 28 | 2 | 38 | 48 | LM, BM | 0 | - | MT- 0/15 | - |
|  |  |  | Conventional scalpel surgery | 15 |  |  |  |  |  | 3 |  | MT- 0/15 |  |
| Jayasooriya et al.  (2019) | Sri Lanka | Retrospective | *Standard Care* | 42 | 73 | 20 | - | 30 (10-72) | BM, LT, FoM | 0 | - | MT- 7/93 for both groups | - |
|  |  |  | Conventional scalpel surgery | 51 |  |  | - |  |  | 16 |  |  |  |
| Yang et al.  (2020) | Taiwan | Retrospective | CO2 laser excision | 69 | 53 | 16 | 71.2±4.9 | 42.5±35.2 | LM, FoM, HP, RA | 23 | 42/42 | MT-8/84 | - |
| Jurczyszyn & Kozakewicz  (2020) | Poland | Prospective | Er:YAG laser vaporization | 32 | 6 | 12 | 60.5±12.9 | 12 | BM, T, AM, P | 11 | - | - | - |
| Romeo et al.  (2020) | Italy | RCT | CO2 laser vaporization w/o margin | 11 | 6 | 5 | 39- 79 | 6 | T, P, BM, G, FoM | 5 | - | - | - |
|  |  |  | CO2 laser vaporization w 3 mm margin | 11 | 4 | 4 | 39- 80 | 6 |  | 4 |  |  |  |
|  |  |  | *Standard Care* | 11 | 4 | 7 | 39- 81 | 6 |  | 8 |  |  |  |
| Yang et al.  (2021) | Taiwan | Retrospective | CO2 laser excision | 111 | 80 | 31 | 51.86±11.85 | 44.9±50.3 | DT, Ventolateral tongue | 15 | 83/28 | MT-4/111 | - |
| Yang et al.  (2021) | Taiwan | Retrospective | CO2 laser excision | 144 | 108 | 36 | 52.17±11.72 | 54.9±54.4 | DT, Ventolateral tongue | 30 | 97/47 | MT-12/144 | - |
| Yang et al.  (2021) | Taiwan | Retrospective | CO2 laser excision | 485 | 412 | 73 | 53.03±11.87 | 65.52±39.6 | BM, T, FoM, RA, HP, G, LM | 149 | 318/167 | MT-32/485 | - |
| Arduino et al.  (2021) | Italy | RCT | Conventional scalpel surgery | 110 | 63 | 47 | 61.1±12.8 | 59.3±27 | T, G, P, BM, LM | 26 | 97/13 | MT- 1/110 | - |
|  |  |  | *Standard Care* | 125 | 54 | 71 | 60.4±13.5 | 59.3±27 |  | 0 | 106/19 | MT- 1/125 | - |
| Yao et al.  (2021) | China | RCT | PDT (AFL + ALA) | 23 | 11 | 12 | 59.9±10 | 12 | LT, FoM, BM | 4 | 17/6 | - | - |
|  |  |  | *AFL* | 21 | 12 | 9 | 58±9.4 | 12 |  | 10 | 15/6 |  |  |
| Georgaki et al.  (2021) | Greece | Retrospective | CO2 laser vaporization | 52 | - | - | 58.3 | 51±23.3 | BM, G, AM, VT, FoM, DT, LT, HP | 13 | - | - | - |
|  |  |  | Conventional scalpel surgery | 45 | - | - | 58.3 | 51±23.3 |  | 25 |  |  |  |
| Gupta et al.  (2021) | India | RCT | CO2 laser vaporization | 10 | 10 | 0 | 25-70 | 3 | - | 2 | 10/0 | - | - |
|  |  |  | Diode laser vaporization | 10 | 10 | 0 |  |  |  | 2 | 10/0 |  |  |
| Petrov et al. (2021) | Bulgaria | RCT | Er:YAG laser vaporization | 36 | 32 | 57 | 56.38±0.97 | 12 | - | 8 | - | - | - |
|  |  |  | Conventional scalpel surgery | 53 |  |  |  |  |  | 8 |  |  |  |
| Sai Praveen et al.  (2021) | India | RCT | Cryosurgery | 15 | - | - | - | 3 | - | 4 | - | - | - |
|  |  |  | Diode laser vaporization | 15 |  |  |  |  |  | 1 |  |  |  |
| Zhou et al.  (2021) | China | Prospective | Conventional scalpel surgery | 27 | 15 | 11 | 46.7 | 26.7 | BM, DT | 2 | - | MT- 0/27 | - |
| Yao et al.  (2022) | China | Retrospective | PDT (AFL + ALA) | 48 | 18 | 30 | 60.7±11.9 | 36 | LT, BM, G, P, DT, FoM | 18 | - | MT- 4/48 | - |
| Campos et al.  (2022) | Brazil | Retrospective | CO2 laser excision | 37 | 14 | 23 | 62.29 | 36(6-239) | FoM, LT, DT, BM, HP, SP, AM | 13 | 15/22 | MT-8/37 |  |
| Rodriguez-Lujan et al.  (2022) | Spain | Prospective | CO2 laser vaporization | 48 | 23 | 25 | 61.3±10 | 18 | G, BM, HP, T, FoM | 27 | 18/30 | MT-1/48 | 18 months- (0.23 ± 0.06)% |
| Vilar-Villaneuva et al.  (2022) | Spain | Retrospective | CO2 laser vaporization | 78 | 22 | 36 | 63.7±13.1 | 57.5±57.9 | FoM, LT, VT, BM, G | 41 | 31/47 | MT- 2/78 | 1 year- 52.9% (SE ±0.061), 3 years- 41.7% (SE ±0.061), 15 years- 26.0% (SE ±0.114) |
| Sagalow et al.  (2022) | USA | Retrospective | Diode laser vaporization | 18 | 5 | 9 | 66.6 | 27.8 | T, BM, HP, SP | 11 | - | - | - |
| de Arruda et al.  (2023) | Brazil | Prospective | Diode laser vaporization | 31 | 6 | 16 | 62.8±12.2 | 28.6±17.3 | T, G, BM, FoM, L | 2 | 27/4 | MT- 0/31 | - |
| Jing et al.  (2024) | China | Prospective | PDT (ALA) | 60 | 25 | 35 | 59.23±12.52 | 13.37±4.44 | L, BM, FoM, DT, VT, HP | 15 | 28/32 | MT-9/60 | - |
| Wang et al.  (2024) | China | Retrospective | PDT (ALA) | 50 | 26 | 24 | 55.5±12.7 | 20±6.9 | FoM, BM, LT, P, G, DT, L | 16 | 16/34 | MT- 2/50 | - |

M- male, F- female, HOL- homogeneous oral leukoplakia, NHOL- non-homogeneous oral leukoplakia, MT- malignant transformation, DFS- disease-free survival; T- Tongue, DT- Dorsal surface of tongue, LT- Lateral margin of tongue, VT- Ventral surface of tongue, BM- buccal mucosa/ cheek, LM- Labial mucosa, L- lip, FoM- Floor of mouth, G- Gingiva, P- Palate, HP- Hard palate, SP- Soft palate, FP- Faucial pillars, RA- Retromolar area, AM- Alveolar mucosa, RCT- randomized controlled trial, SE- standard error.

**Appendix Table 2:** Evaluation of the certainty of evidence following the GRADE guidelines for the proportion of oral leukoplakia (OL) recurrence based on surgical techniques

| **No. of studies** | **Certainty assessment** | | | | | | **Effect** | | | **Certainty** | **Importance** |
| --- | --- | --- | --- | --- | --- | --- | --- | --- | --- | --- | --- |
|  | **Study design** | **Risk of bias** | **Inconsistency** | **Indirectness** | **Imprecision** | **Other considerations** | **No. of events** | **No. of individuals** | **Rate**  **(95% CI)** |  |  |
| **CO_2_ laser excision** | | | | | | | | | | | |
| 14 | observational studies | serious^a^ | serious^b^ | not serious | not serious | publication bias strongly suspected  all plausible residual confounding would reduce the demonstrated effect | 348 | 1487 | 19 per 100 (14 to 24) | ⨁⨁◯◯ Low | IMPORTANT |
| **CO_2_ laser excision plus vaporization** | | | | | | | | | | | |
| 5 | observational studies | serious^a^ | serious^b^ | not serious | not serious | publication bias strongly suspected  all plausible residual confounding would reduce the demonstrated effect | 397 | 2511 | 11 per 100 (6 to 17) | ⨁⨁◯◯ Low | IMPORTANT |
| **CO_2_ laser vaporization** | | | | | | | | | | | |
| 21 | observational studies | serious^a^ | serious^b^ | not serious | not serious | publication bias strongly suspected  all plausible residual confounding would reduce the demonstrated effect | 280 | 1118 | 27 per 100 (21 to 34) | ⨁⨁◯◯ Low | IMPORTANT |
| **Conventional surgery (scalpel)** | | | | | | | | | | | |
| 18 | observational studies | serious^a^ | serious^b^ | not serious | not serious | publication bias strongly suspected  all plausible residual confounding would reduce the demonstrated effect | 248 | 982 | 24 per 100 (17 to 32) | ⨁⨁◯◯ Low | IMPORTANT |
| **Cryosurgery** | | | | | | | | | | | |
| 7 | observational studies | serious^a^ | serious^b^ | not serious | not serious | publication bias strongly suspected  all plausible residual confounding would reduce the demonstrated effect | 35 | 159 | 21 per 100 (9 to 33) | ⨁⨁◯◯ Low | IMPORTANT |
| **Diode laser vaporization** | | | | | | | | | | | |
| 10 | observational studies | serious^a^ | serious^b^ | not serious | not serious | publication bias strongly suspected  all plausible residual confounding would reduce the demonstrated effect | 38 | 220 | 14 per 100 (4 to 24) | ⨁⨁◯◯ Low | IMPORTANT |
| **Er:YAG laser vaporization** | | | | | | | | | | | |
| 6 | observational studies | serious^a^ | serious^b^ | not serious | not serious | publication bias strongly suspected  all plausible residual confounding would reduce the demonstrated effect | 69 | 189 | 31 per 100 (9 to 53) | ⨁⨁◯◯ Low | NOT IMPORTANT |
| **Nd:YAG laser excision** | | | | | | | | | | | |
| 2 | observational studies | serious^a^ | not serious | not serious | not serious | publication bias strongly suspected  all plausible residual confounding would reduce the demonstrated effect | 11 | 36 | 30 per 100 (15 to 45) | ⨁⨁⨁◯ Moderate | NOT IMPORTANT |
| **Nd:YAG laser vaporization** | | | | | | | | | | | |
| 5 | observational studies | serious^a^ | serious^b^ | not serious | not serious | publication bias strongly suspected  all plausible residual confounding would reduce the demonstrated effect | 35 | 168 | 18 (5 to 31) | ⨁⨁◯◯ Low | NOT IMPORTANT |
| **PDT** | | | | | | | | | | | |
| 8 | observational studies | serious^a^ | serious^b^ | not serious | not serious | publication bias strongly suspected  all plausible residual confounding would reduce the demonstrated effect | 73 | 386 | 19 per 100 (10 to 28) | ⨁⨁◯◯ Low | IMPORTANT |

**CI:** confidence interval; **PDT:** photodynamic therapy

a. potential bias in included studies

b. high heterogeneity among included studies

**Appendix Table 3:** Evaluation of the certainty of evidence following the GRADE guidelines for the risk of malignant transformation (MT) of oral leukoplakia (OL) based on the recurrence status

| **Certainty assessment** | | | | | | | **No. of patients** | | **Effect** | | **Certainty** | **Importance** |
| --- | --- | --- | --- | --- | --- | --- | --- | --- | --- | --- | --- | --- |
| **No. of studies** | **Study design** | **Risk of bias** | **Inconsistency** | **Indirectness** | **Imprecision** | **Other considerations** | **Recurrent OL** | **Non-recurrent OL** | **Relative (95% CI)** | **Absolute (95% CI)** |  |  |
| **MT of OL based on the recurrence status** | | | | | | | | | | | | |
| 6 | observational studies | serious^a^ | not serious | not serious | not serious | very strong association  all plausible residual confounding would reduce the demonstrated effect | 30/150 (20.0%) | 13/443 (2.9%) | **RR 7.39** (3.90 to 14.02) | **188 more per 1,000** (from 85 more to 382 more) | ⨁⨁⨁⨁  High | CRITICAL |

#### CI: confidence interval; RR: risk ratio

a. potential bias in included studies

**Appendix:** **References to the included studies**

1. Arduino PG, Cafaro A, Cabras M, Gambino A, Broccoletti R. 2018. Treatment outcome of oral leukoplakia with er:Yag laser: A 5-year follow-up prospective comparative study. Photomed Laser Surg. 36(12):631-633.

2. Arduino PG, Lodi G, Cabras M, Macciotta A, Gambino A, Conrotto D, Karimi D, Haddad GE, Carbone M, Broccoletti R. 2021. A randomized controlled trial on efficacy of surgical excision of nondysplastic leukoplakia to prevent oral cancer. Cancer Prev Res (Phila). 14(2):275-284.

3. Arora KS, Bansal R, Mohapatra S, Verma A, Sharma S, Pareek S. 2018. Prevention of malignant transformation of oral leukoplakia and oral lichen planus using laser: An observational study. Asian Pac J Cancer Prev. 19(12):3635-3641.

4. Brouns ER, Baart JA, Karagozoglu KH, Aartman IH, Bloemena E, van der Waal I. 2013. Treatment results of co2 laser vaporisation in a cohort of 35 patients with oral leukoplakia. Oral Dis. 19(2):212-216.

5. Campos WG, Esteves CV, Gallo CB, Domaneschi C, Aranha ACC, Lemos CA. 2022. Treatment of oral leukoplakia with co2 laser (10,600 nm): Analysis of 37 cases. Braz Oral Res. 36:e014.

6. Chainani-Wu N, Lee D, Madden E, Sim C, Collins K, Silverman S, Jr. 2015. Clinical predictors of oral leukoplakia recurrence following co₂ laser vaporization. J Craniomaxillofac Surg. 43(9):1875-1879.

7. Chandu A, Smith AC. 2005. The use of co2 laser in the treatment of oral white patches: Outcomes and factors affecting recurrence. Int J Oral Maxillofac Surg. 34(4):396-400.

8. Chiesa F, Boracchi P, Tradati N, Rossi N, Costa L, Giardini R, Marazza M, Zurrida S. 1993. Risk of preneoplastic and neoplastic events in operated oral leukoplakias. Eur J Cancer B Oral Oncol. 29b(1):23-28.

9. Chiesa F, Tradati N, Sala L, Costa L, Podrecca S, Boracchi P, Bandieramonte G, Mauri M, Molinari R. 1990. Follow-up of oral leukoplakia after carbon dioxide laser surgery. Arch Otolaryngol Head Neck Surg. 116(2):177-180.

10. Chiu CT, Li CF, Li JR, Wang J, Chuang CY, Chiang WF, Huang SC, Chang SW. 2011. Candida invasion and influences in smoking patients with multiple oral leucoplakias--a retrospective study. Mycoses. 54(5):e377-383.

11. Chu FW, Silverman S, Jr., Dedo HH. 1988. Co2 laser treatment of oral leukoplakia. Laryngoscope. 98(2):125-130.

12. Cloitre A, Rosa RW, Arrive E, Fricain JC. 2018. Outcome of co2 laser vaporization for oral potentially malignant disorders treatment. Medicina Oral Patologia Oral y Cirugia Bucal. 23(2):e237-e247.

13. Das S, Mohammad S, Singh V, Gupta S. 2015. Neodymium:Yttrium aluminum garnet laser in the management of oral leukoplakia: A case series. Contemp Clin Dent. 6(Suppl 1):S32-35.

14. de Arruda JAA, Silva LVO, Kato C, Pinheiro JJV, Abreu LG, Silva TA, Ferreira MVL, Souza LN, Mesquita RA. 2023. Management of oral leukoplakia with an 808-nm high-power diode laser: A single-center experience. Lasers Med Sci. 38(1):141.

15. Del Corso G, Gissi DB, Tarsitano A, Costabile E, Marchetti C, Montebugnoli L, Foschini MP. 2015. Laser evaporation versus laser excision of oral leukoplakia: A retrospective study with long-term follow-up. J Craniomaxillofac Surg. 43(6):763-768.

16. Deppe H, Hillemanns M, Hauck W. 2004. Recurrence rates of premalignant lesions after co2 laser vaporization. Medical Laser Application. 19(1):55-60.

17. Deppe H, Mücke T, Hohlweg-Majert B, Hauck W, Wagenpfeil S, Hölzle F. 2012. Different co2 laser vaporization protocols for the therapy of oral precancerous lesions and precancerous conditions: A 10-year follow-up. Lasers Med Sci. 27(1):59-63.

18. Frame JW, Das Gupta AR, Dalton GA, Rhys Evans PH. 1984. Use of the carbon dioxide laser in the management of premalignant lesions of the oral mucosa. J Laryngol Otol. 98(12):1251-1260.

19. Gabrić D, Brailo V, Ivek A, Krpan K, Matulić N, Vrdoljak DV, Baraba A, Vučićević Boras V. 2019. Evaluation of innovative digitally controlled er:Yag laser in surgical treatment of oral leukoplakia - a preliminary study. Acta Clin Croat. 58(4):615-620.

20. Galletta V, Azevedo L, Lodi G, Migliari D. 2017. Factors affecting clinical outcomes after treatment of oral leukoplakia with co(2) and diode laser. J Contemp Dent Pract. 18(9):775-780.

21. Georgaki M, Avgoustidis D, Theofilou VI, Piperi E, Pettas E, Kalyvas DG, Vlachodimitropoulos D, Perisanidis C, Lazaris AC, Nikitakis NG. 2021. Recurrence in oral premalignancy: Clinicopathologic and immunohistochemical analysis. Diagnostics (Basel). 11(5).

22. Gooris PJ, Roodenburg JL, Vermey A, Nauta JM. 1999. Carbon dioxide laser evaporation of leukoplakia of the lower lip: A retrospective evaluation. Oral Oncol. 35(5):490-495.

23. Gupta A, Pagare SS, Bhatnagar S, Waghmare M, Vahanwale S. 2021. Comparative evaluation of the efficacy of co2 and diode laser in the treatment outcome of oral homogeneous leukoplakia. Journal of Indian Academy of Oral Medicine and Radiology. 33(4):447-452.

24. Hamadah O, Thomson PJ. 2009. Factors affecting carbon dioxide laser treatment for oral precancer: A patient cohort study. Lasers Surg Med. 41(1):17-25.

25. Holmstrup P, Vedtofte P, Reibel J, Stoltze K. 2006. Long-term treatment outcome of oral premalignant lesions. Oral Oncol. 42(5):461-474.

26. Horch HH, Gerlach KL. 1982. Co2 laser treatment of oral dysplastic precancerous lesions: A preliminary report. Lasers Surg Med. 2(2):179-185.

27. Horch HH, Gerlach KL, Schaefer HE. 1986. Co2 laser surgery of oral premalignant lesions. Int J Oral Maxillofac Surg. 15(1):19-24.

28. Ishii J, Fujita K, Munemoto S, Komori T. 2004. Management of oral leukoplakia by laser surgery: Relation between recurrence and malignant transformation and clinicopathological features. J Clin Laser Med Surg. 22(1):27-33.

29. Jayasooriya PR, Dayaratne K, Dissanayake UB, Warnakulasuriya S. 2020. Malignant transformation of oral leukoplakia: A follow-up study. Clin Oral Investig. 24(12):4563-4569.

30. Jerjes W, Upile T, Hamdoon Z, Al-Khawalde M, Morcos M, Mosse CA, Hopper C. 2012. Co2 laser of oral dysplasia: Clinicopathological features of recurrence and malignant transformation. Lasers Med Sci. 27(1):169-179.

31. Jerjes W, Upile T, Hamdoon Z, Mosse CA, Akram S, Hopper C. 2011. Photodynamic therapy outcome for oral dysplasia. Lasers Surg Med. 43(3):192-199.

32. Jing Y, Shu R, Wu T, Liu D, Luo X, Sun J, Chen F. 2024. Clinical efficacy of photodynamic therapy of oral potentially malignant disorder. Photodiagnosis Photodyn Ther. 46.

33. Jurczyszyn K, Kozakiewicz M. 2020. Application of texture and fractal dimension analysis to estimate effectiveness of oral leukoplakia treatment using an er:Yag laser-a prospective study. Materials (Basel). 13(16).

34. Kawczyk-Krupka A, Waśkowska J, Raczkowska-Siostrzonek A, Kościarz-Grzesiok A, Kwiatek S, Straszak D, Latos W, Koszowski R, Sieroń A. 2012. Comparison of cryotherapy and photodynamic therapy in treatment of oral leukoplakia. Photodiagnosis Photodyn Ther. 9(2):148-155.

35. Kharadi UA, Onkar S, Birangane R, Chaudhari S, Kulkarni A, Chaudhari R. 2015. Treatment of oral leukoplakia with diode laser: A pilot study on indian subjects. Asian Pac J Cancer Prev. 16(18):8383-8386.

36. Kuribayashi Y, Tsushima F, Sato M, Morita K, Omura K. 2012. Recurrence patterns of oral leukoplakia after curative surgical resection: Important factors that predict the risk of recurrence and malignancy. J Oral Pathol Med. 41(9):682-688.

37. Matsumoto K, Suzuki H, Asai T, Wakabayashi R, Enomoto Y, Kitayama M, Shigeoka M, Kimoto A, Takeuchi J, Yutori H et al. 2015. Clinical investigation of carbon dioxide laser treatment for lingual leukoplakia. Journal of Oral and Maxillofacial Surgery, Medicine, and Pathology. 27(4):493-497.

38. Matulić N, Bago I, Sušić M, Gjorgievska E, Kotarac Knežević A, Gabrić D. 2019. Comparison of er:Yag and er,cr:Ysgg laser in the treatment of oral leukoplakia lesions refractory to the local retinoid therapy. Photobiomodul Photomed Laser Surg. 37(6):362-368.

39. Mogedas-Vegara A, Hueto-Madrid JA, Chimenos-Küstner E, Bescós-Atín C. 2015. The treatment of oral leukoplakia with the co2 laser: A retrospective study of 65 patients. J Craniomaxillofac Surg. 43(5):677-681.

40. Montebugnoli L, Frini F, Gissi DB, Gabusi A, Cervellati F, Foschini MP, Marchetti C. 2012. Histological and immunohistochemical evaluation of new epithelium after removal of oral leukoplakia with nd:Yag laser treatment. Lasers Med Sci. 27(1):205-210.

41. Monteiro L, Barbieri C, Warnakulasuriya S, Martins M, Salazar F, Pacheco JJ, Vescovi P, Meleti M. 2017. Type of surgical treatment and recurrence of oral leukoplakia: A retrospective clinical study. Med Oral Patol Oral Cir Bucal. 22(5):e520-e526.

42. Nammour S, Zeinoun T, Namour A, Vanheusden A, Vescovi P. 2017. Evaluation of different laser-supported surgical protocols for the treatment of oral leukoplakia: A long-term follow-up. Photomed Laser Surg. 35(11):629-638.

43. Natekar M, Raghuveer HP, Rayapati DK, Shobha ES, Prashanth NT, Rangan V, Panicker AG. 2017. A comparative evaluation: Oral leukoplakia surgical management using diode laser, co2 laser, and cryosurgery. J Clin Exp Dent. 9(6):e779-e784.

44. Pandey M, Thomas G, Somanathan T, Sankaranarayanan R, Abraham EK, Jacob BJ, Mathew B. 2001. Evaluation of surgical excision of non-homogeneous oral leukoplakia in a screening intervention trial, kerala, india. Oral Oncol. 37(1):103-109.

45. Petrov PG, Daskalov HI, Dinkova AS. 2021. Randomized comparative study in the treatment of oral leucoplakia with laser and conventional surgery. Braz Dent Sci. 24(4):1-9.

46. Pindborg JJ, Jolst O, Renstrup G, Roed-Petersen B. 1968. Studies in oral leukoplakia: A preliminary report on the period pervalence of malignant transformation in leukoplakia based on a follow-up study of 248 patients. J Am Dent Assoc. 76(4):767-771.

47. Prasad M, Kale TP, Halli R, Kotrashetti SM, Baliga SD. 2009. Liquid nitrogen cryotherapy in the management of oral lesions: A retrospective clinical study. J Maxillofac Oral Surg. 8(1):40-42.

48. Reddy Kundoor VK, Patimeedi A, Roohi S, Maloth KN, Kesidi S, Masabattula GK. 2015. Efficacy of diode laser for the management of potentially malignant disorders. J Lasers Med Sci. 6(3):120-123.

49. Ribeiro AS, de Aguiar MC, do Carmo MA, de Abreu MH, Silva TA, Mesquita RA. 2011. 660 asgaal laser to alleviate pain caused by cryosurgical treatment of oral leukoplakia: A preliminary study. Photomed Laser Surg. 29(5):345-350.

50. Rodriguez-Lujan A, López-Jornet P, Pons-Fuster López E. 2022. Recurrence of oral leukoplakia after co(2) laser resection: A prospective longitudinal study. Cancers (Basel). 14(21).

51. Romeo U, Mohsen M, Palaia G, Bellisario A, Del Vecchio A, Tenore G. 2020. Co2 laser ablation of oral leukoplakia: With or without extension of margins? Clin Ter. 171(3):e209-e215.

52. Roodenburg JLN, Panders AK, Vermey A. 1991. Carbon dioxide laser surgery of oral leukoplakia. Oral Surgery, Oral Medicine, Oral Pathology. 71(6):670-674.

53. Sagalow ES, Kumar AT, Banoub RG, Xiao KB, Zhan T, Luginbuhl A, Curry JM. 2022. Recurrence of premalignant oral cavity and oropharynx lesions after pulsed diode laser treatment. Am J Otolaryngol. 43(5):103556.

54. Sai Praveen KNV, Veeraraghavan G, Ramesh T, Teja T, Upendra G, Kalyan Y. 2021. Efficacy of cryogun versus diode laser therapy in the management of oral leukoplakia. Journal of Indian Academy of Oral Medicine and Radiology. 33(1):60-65.

55. Saibene AM, Rosso C, Castellarin P, Vultaggio F, Pipolo C, Maccari A, Ferrari D, Abati S, Felisati G. 2019. Managing benign and malignant oral lesions with carbon dioxide laser: Indications, techniques, and outcomes for outpatient surgery. Surg J (N Y). 5(3):e69-e75.

56. Sako K, Marchetta FC, Hayes RL. 1972. Cryotherapy of intraoral leukoplakia. Am J Surg. 124(4):482-484.

57. Schoelch ML, Sekandari N, Regezi JA, Silverman S, Jr. 1999. Laser management of oral leukoplakias: A follow-up study of 70 patients. Laryngoscope. 109(6):949-953.

58. Schwarz F, Maraki D, Yalcinkaya S, Bieling K, Böcking A, Becker J. 2005. Cytologic and DNA-cytometric follow-up of oral leukoplakia after co2- and er:Yag-laser assisted ablation: A pilot study. Lasers Surg Med. 37(1):29-36.

59. Selvam NP, Sadaksharam J, Singaravelu G, Ramu R. 2015. Treatment of oral leukoplakia with photodynamic therapy: A pilot study. J Cancer Res Ther. 11(2):464-467.

60. Sieroń A, Adamek M, Kawczyk-Krupka A, Mazur S, Ilewicz L. 2003. Photodynamic therapy (pdt) using topically applied delta-aminolevulinic acid (ala) for the treatment of oral leukoplakia. J Oral Pathol Med. 32(6):330-336.

61. Silverman S, Jr., Gorsky M, Lozada F. 1984. Oral leukoplakia and malignant transformation. A follow-up study of 257 patients. Cancer. 53(3):563-568.

62. Sundberg J, Korytowska M, Holmberg E, Bratel J, Wallström M, Kjellström E, Blomgren J, Kovács A, Öhman J, Sand L et al. 2019. Recurrence rates after surgical removal of oral leukoplakia-a prospective longitudinal multi-centre study. PLoS One. 14(12):e0225682.

63. Thomas G, Kunnambath R, Somanathan T, Mathew B, Pandey M, Rangaswamy S. 2012. Long-term outcome of surgical excision of leukoplakia in a screening intervention trial, kerala, india. Journal of Indian Academy of Oral Medicine and Radiology. 24(2):126-129.

64. Thomson PJ, Wylie J. 2002. Interventional laser surgery: An effective surgical and diagnostic tool in oral precancer management. Int J Oral Maxillofac Surg. 31(2):145-153.

65. van der Hem PS, Nauta JM, van der Wal JE, Roodenburg JL. 2005. The results of co2 laser surgery in patients with oral leukoplakia: A 25 year follow up. Oral Oncol. 41(1):31-37.

66. Vedtofte P, Holmstrup P, Hjørting-Hansen E, Pindborg JJ. 1987. Surgical treatment of premalignant lesions of the oral mucosa. Int J Oral Maxillofac Surg. 16(6):656-664.

67. Vilar-Villanueva M, Somoza-Martín JM, Blanco-Carrión A, García-García A, García-Carnicero T, Marichalar-Mendía X, Gallas-Torreira M, Gándara-Vila P. 2022. Importance of the vaporization margin during co(2) laser treatment of oral leukoplakia: A survival study. Oral Dis.

68. Vivek V, Jayasree RS, Balan A, Sreelatha KT, Gupta AK. 2008. Three-year follow-up of oral leukoplakia after neodymium:Yttrium aluminum garnet (nd:Yag) laser surgery. Lasers in Medical Science. 23(4):375-379.

69. Vlk M, Smucler R. 2013. The efficacy of diode laser removal of leukoplakias on the tongue and in lower buccal cavity compared to other buccal cavity locations. A two-year study. Central European Journal of Medicine. 8(2):192-203.

70. Wang Y, Tang H, Wang K, Zhao Y, Xu J, Fan Y. 2024. Clinical evaluation of photodynamic therapy for oral leukoplakia: A retrospective study of 50 patients. BMC Oral Health. 24(1).

71. White JM, Chaudhry SI, Kudler JJ, Sekandari N, Schoelch ML, Silverman S, Jr. 1998. Nd:Yag and co2 laser therapy of oral mucosal lesions. J Clin Laser Med Surg. 16(6):299-304.

72. Yang SW, Lee YS, Chang LC, Hwang CC, Chen TA. 2020. Clincopathological characteristics and treatment outcomes of oral leukoplakia by carbon dioxide laser excision in the elderly patients. Head Neck. 42(5):1014-1023.

73. Yang SW, Lee YS, Chang LC, Yang CH, Luo CM. 2021. An anatomical perspective on clinicopathological characteristics and treatment outcomes of dorsal and ventrolateral tongue leukoplakia after carbon dioxide laser surgery. BMC Oral Health. 21(1):45.

74. Yang SW, Lee YS, Chang LC, Yang CH, Luo CM, Wu PW. 2021. Oral tongue leukoplakia: Analysis of clinicopathological characteristics, treatment outcomes, and factors related to recurrence and malignant transformation. Clin Oral Investig. 25(6):4045-4058.

75. Yang SW, Lee YS, Wu PW, Chang LC, Hwang CC. 2021. A retrospective cohort study of oral leukoplakia in female patients-analysis of risk factors related to treatment outcomes. Int J Environ Res Public Health. 18(16).

76. Yang SW, Tsai CN, Lee YS, Chen TA. 2011. Treatment outcome of dysplastic oral leukoplakia with carbon dioxide laser--emphasis on the factors affecting recurrence. J Oral Maxillofac Surg. 69(6):e78-87.

77. Yao Y, Shi L, Wang Y, Shen X, Ye S, Tang G, Wu L. 2021. Ablative fractional laser-assisted photodynamic therapy vs. Ablative fractional laser for oral leukoplakia treatment: A randomized, controlled pilot study. Photodiagnosis Photodyn Ther. 36:102523.

78. Yao YL, Wang YF, Li CX, Wu L, Tang GY. 2022. Management of oral leukoplakia by ablative fractional laser-assisted photodynamic therapy: A 3-year retrospective study of 48 patients. Lasers Surg Med. 54(5):682-687.

79. Yasmeen SA, Satheesha Reddy BH, Ramesh MV, Birajdar SS, Yarram A, Kumar NN. 2019. A comparative evaluation of diode laser ablation versus scalpel excision for management of oral leukoplakia. Journal of Indian Academy of Oral Medicine and Radiology. 31(2):94-99.

80. Zhou B, Yuan KF, Chen WL. 2021. Use of allograft dermal matrix for repairing large oral epithelial defects: Outcomes of patients with lingual and buccal leukoplakia. J Cosmet Dermatol. 20(9):2753-2757.
